# GreenVMAS: Virtual Organization Based Platform for Heating Greenhouses Using Waste Energy from Power Plants

**DOI:** 10.3390/s18030861

**Published:** 2018-03-14

**Authors:** Alfonso González-Briones, Pablo Chamoso, Hyun Yoe, Juan M. Corchado

**Affiliations:** 1 BISITE Digital Innovation Hub, University of Salamanca, Edificio Multiusos I+D+i, Calle Espejo 2, 37007 Salamanca, Spain; alfonsogb@usal.es (A.G.-B.); corchado@usal.es (J.M.C.); 2 School of Information and Communication Engineering, Sunchon National University, Suncheon 540-742, Korea; yhyun@sunchon.ac.kr; 3 Department of Electronics, Information and Communication, Faculty of Engineering, Osaka Institute of Technology, Osaka 535-8585, Japan; 4 Pusat Komputeran dan Informatik, Universiti Malaysia Kelantan, Karung Berkunci 36, Pengkaan Chepa, Kota Bharu 16100, Kelantan, Malaysia

**Keywords:** waste energy reuse, power plant, greenhouse, context awareness, case-based reasoning, virtual organizations

## Abstract

The gradual depletion of energy resources makes it necessary to optimize their use and to reuse them. Although great advances have already been made in optimizing energy generation processes, many of these processes generate energy that inevitably gets wasted. A clear example of this are nuclear, thermal and carbon power plants, which lose a large amount of energy that could otherwise be used for different purposes, such as heating greenhouses. The role of GreenVMAS is to maintain the required temperature level in greenhouses by using the waste energy generated by power plants. It incorporates a case-based reasoning system, virtual organizations and algorithms for data analysis and for efficient interaction with sensors and actuators. The system is context aware and scalable as it incorporates an artificial neural network, this means that it can operate correctly even if the number and characteristics of the greenhouses participating in the case study change. The architecture was evaluated empirically and the results show that the user’s energy bill is greatly reduced with the implemented system.

## 1. Introduction

The optimization of energy use is currently a very important field of research [1]. However, the use of the waste energy that is produced as a result of these processes is just as important [2]. The majority of research proposals in this area are focused on the reuse of waste thermal energy. In a study conducted by Karellas et al. [3], exhaust gases are used in a cement plant as a way of obtaining energy. Bianchi [4] also makes use of waste heat at power plants by using thermodynamic cycles based on carbon dioxide in supercritical phase (sCO_2_). Reviews of the state of the art in the use of thermal energy conclude that with the use of existing technologies, 20% to 50% of the total residual heat can be collected and reused in other activities [5].

Nuclear power plants produce the greatest amounts of waste thermal energy. The nuclear reactor creates and controls atomic emission, generating a large amount of thermal energy (heat). This heat turns water into steam, at different levels of pressure and temperature. Due to the change in temperature to which it is subjected, high-temperature steam leaves the containment building. It reaches the turbine causing it to turn, in this way thermal energy is converted into kinetic energy. This turbine is connected to an electric generator, converting this kinetic energy into electrical energy. Although energy conversion processes are quite optimized, a lot of waste thermal energy is produced in the process of converting it into kinetic energy. This thermal energy could be used in other activities.

The cost of growing crops in a greenhouse is very high because specific conditions have to be maintained within it for the proper development of the crop. The cost of produce increases when outside temperature is low/high; this is because more money will be spent on electricity and gas for heating/cooling the greenhouse. Since greenhouses are costly and require large amounts of energy they are ideal for making use of the residual energy of power plant processes. We should also point to the large greenhouse areas that could benefit from such a system, such as those found in the southwestern region of Korea (South Jeolla). Due to the orography and climate conditions of the region, it is an ideal place for growing rice, wheat, barley, beans and potatoes. However, to create optimal growing conditions for the crops, they still have to be cultivated in greenhouses with heating systems. In this way, low temperature stress in crops is avoided, having positive effects on the yield and the quality of the produce.

One of the main approaches for efficient energy use is based on recovering and recycling the power plants’ energy waste. Waste heat recovery units help to recover heat from hot streams with potential high energy content. This waste is produced as a result of different processes that take place at power plants, in [6] waste heat is recovered in the form of hot water. The present work exploits the hot water obtained from the waste heat produced by different power plant processes. Multiple parameters are considered when distributing the energy to greenhouses, in this way the desired temperature is established effectively.

Since it would be very costly to deploy the proposed system at a real power plant, the present work simulates real conditions in order to evaluate and determine the viability of the proposed system. The waste energy information was provided by the power plant in order to make the simulation as real as possible, to calculate what energy savings could be achieved in greenhouses if the infrastructure were deployed. The purpose of the infrastructure is to distribute energy among several greenhouses which belong to the Sunchon National University and to the University of Salamanca.

However, to distribute energy efficiently and guarantee equal treatment for all the greenhouses involved in the case study, information on them has to be gathered and evaluated first so that the system can adapt to their needs autonomously and effectively. Ambient technology [7] is ideal for the needs of the system as it adapts technology to people. In this case, it allows the system to respond intelligently to the needs and requests of greenhouse farmers, easing their daily tasks. GreenVMAS has been developed as a distributed, Organization based Multi-Agent System (MAS) [8], this architecture is essential for achieving common goals within an ambient intelligence framework. By integrating intelligent and dynamic mechanisms that have the ability to learn from past experiences, the proposed architecture provides users with tools that save energy efficiently.

More specifically, this work develops a Virtual Organization based MAS. It is aimed at improving the way in which heat water from waste heat recovery systems is used and distributed among a set of greenhouses. The system is based on PANGEA [9], which is a high-level Multi-Agent Platform based on Virtual Organizations (VO) for intelligent Information Fusion (IF) and management; in addition, it defines rules and services within the system.

GreenVMAS integrates different elements and functionalities. The developed VO-MAS provides the basis for the analysis and transformation of data. Decision-making and data processing tasks are carried out by autonomous agents that implement different algorithms and exchange information between each other. The agents incorporate an Artificial Neural Network (ANN) to predict the energy requirements of each greenhouse in order to establish the target temperature. Moreover, different sensors are integrated in the system to gather all the required data and send them to the platform before they are fused. Finally, the system includes a software platform that allows the farmers to communicate with their greenhouses. This includes real-time status monitoring, a new pattern definition that agents have to consider when analyzing data and distributing water, or statistical information and data sheets with consumption and efficiency information.

The rest of the article is structured as follows: Section 2 reviews related state of the art projects and most commonly used technologies. Section 3 describes the GreenVMAS system. Section 4 outlines the case studies that were performed to evaluate the proposed system. Section 5 outlines the results of the evaluated scenarios. Finally, Section 6 draws conclusions from this proposal and discusses future lines of work.

## 2. Related Work

This section presents a review of the state of the art. The latest proposals in the field of waste energy recovery are described and the technologies used in these systems are analysed.

### 2.1. Recent Projects

The emergence of new technologies gave rise to projects that focus on improving our interaction with different types of environments. One specific application is the conservation of energy; this can be observed in the following projects:

- The European GASTone project [10]: New powertrain concept based on the integration of energy recovery, storage and reuse using an engine system and control strategies, its energy recovery strategy is focused on two aspects: (i) the recovery of a part of the generated kinetic energy by adapting a belt driven generator; (ii) recovery of waste heat with an energy cascading approach: thermoelectric generator operating at high temperature. The system includes an energy storage system and electrified auxiliaries such as coolant and oil pumps, auxiliary turbo charger and air conditioning compressors. The project aims to achieve economic savings of 50%.
- The European TRIPOD project [11]: proposed the development and validation of a new propulsion concept for improved energy efficiency in ships. Currently, most cargo ship propellers waste about 40 percent of energy in the form of rotational losses in the wake, vortex generation, noise production, cavitation, etc.
- The European HEATRECAR project [12]: aims to reduce energy consumption and curb CO_2_ emissions of vehicles by massively harvesting electrical energy from the heat within the exhaust system and reuse this energy to supply electrical components within the vehicle or to feed the powertrain of hybrid electrical vehicles (HEV). Thermal energy is recovered by novel, laboratory-available thermoelectric materials which can work at high temperatures and exhibit high performance.
- European Intensified Heat Transfer Technologies for Enhanced Heat Recovery (INTHEAT) [13]: this project aims to achieve 20% to 30% energy savings in energy recovery systems, through: (i) Enhancing our understanding of heat exchange and waste heat recovery; (ii) combining an innovative heat transfer design to achieve the synergy of separate novel technologies with focus on conventional, plate-fin and membrane exchangers. Although the current trends in heat exchangers have a great impact on industrial processes, the traditional heat exchangers based on tubular constructions, shell and tubes and air cooled exchangers will still be used; (iii) this project proposes new materials to improve economic and environmental performance of heat transfer media (with focus on advanced heat transfer fluids); (iv) the implementation of the developed technologies in heat exchanger networks (HENs) through the integration of intelligent processes and control techniques.

Table 1 outlines the similarities and differences between the projects as well as their advantages and disadvantages, comparing also our GreenVMAS proposal. As can be seen, the main focus of all these projects is the reuse of energy. However, to provide real solutions these proposals require prior modifications. Moreover, they emit pollutants, they do not reduce the consumption of electricity or the computational load prevents them from becoming highly optimized. Our proposal, on the other hand, does not require any modification (but rather the addition of a complementary infrastructure), its operation does not produce polluting substances (it reduces CO_2_ emissions). Furthermore, it successfully reduces the consumption of energy and does not require a large computing load.

Other recent research focusses on obtaining hot water from flue gases [6]; the study develops a new waste heat and water recovery technology.

The importance of greenhouse farming lies in the increasing demand for agricultural products which is caused by population growth [14,15]. So far, greenhouse climate control has been improved only by using feedback-feed-forward control systems in combination with control algorithms and on-line adaptation of model parameter [16,17], however the issue of energy efficiency in greenhouses is only a recent concern. Recent research has focused on measuring the efficiency of energy use in greenhouses [18] and on the evaluation of using various renewable energy sources for heating a greenhouse [14], but there are no projects in the state of the art that would use the energy wastes produced by other industries.

In this research work, new energy recovery technologies are used to distribute the waste energy generated by power plants to greenhouses. The aim is to optimize the use of energy and thus, decrease the costs involved in maintaining a greenhouse. To this end, IF and rule-based reasoning techniques were used. Multiple sensors obtain data that is uncertain, inaccurate or may even contain conflicting information [19], thus all the data gathered from different sensors must be fused. One of the most commonly used solutions to IF problems is the use of multi-agent architectures, especially because they can implement new algorithms for IF and manage high level information [20,21,22]. An automated reasoning system is implemented [23] to provide the system with the ability to make decisions based on new and past information obtained by a network of heterogeneous sensors. As for data fusion, situation refinement and threat detection are controlled by knowledge-based methods like the one mentioned before [18].

### 2.2. Review of Artificial Intelligence Techniques for Context Awareness

To optimize the distribution of the waste energy that is recovered from power stations, apart from the sensor network and actuators that monitor and control the power distribution system, artificial intelligence techniques need to be employed. This is because energy must be distributed in such a way that all the recovered energy is used equitably by all the participants. AI techniques allow the calculation of the amount of energy required by each participant within the system.

Artificial intelligence techniques are employed for this purpose because they allow the system to learn from past situations. This permits the system to adapt even to external parameters that influence the system, such as individual attendee losses.

One of the most widespread artificial intelligence techniques that allows systems to learn from past situations are CBR systems [24]. CBR systems find solutions to new problems by considering and adapting old solutions stored in the case base. They apply a reasoning process to previous cases to interpret a new situation or create an equitable solution to a new problem [25]. The main CBR process involves the cases that define a problem, the solution and the final result. In addition, when the system receives a new case, that case is solved through the reasoning cycle which consists of four stages: recovery, reuse, revise and retain.

ANNs are used in numerous fields to predict from a series of input parameters. For example, ANN can be applied to classify patterns [26], make clusters [27], or perform predictive, optimization and control tasks [28]. This work uses supervised learning neural networks for prediction, a well-known neural network is Multi-Layer Perceptron (MLP) [29]. Long short-term memory neural networks (LSTM) [30] include the radial basis function (RBF) [31] or recurrent neural networks. ANN has been used for years now in electric applications, primarily to estimate different parameters (the customers’ demand, the consumption, the processing time, the maintenance dates, etc.), predict failures and risks or traffic flows [32]. ANN was used in this work to design a predictive maintenance model, ANNs are commonly used for this purpose due to their efficacy in this area [33]. In addition to neural networks, there are other models that can be employed in prediction systems, such as Support Vector Regression (SVR) or different linear and nonlinear models. Although these models are not suitable for generalised applications, they are applicable to certain case studies [34,35]. In this work we opt for MLP, this is because these networks are capable of approximating any function accurately by a single hidden layer, as stated by Kolmogorv. In addition, we introduce restrictions on the number of hidden layer neurons and the functions of activation and transfer. However, these activation and transfer functions were not meant for calculating the approximate as they provide inaccurate results. Generic functions are used instead.

Agent-based technology is widely used in artificial intelligence systems because it facilitates the implementation of AI techniques, especially those that allow the system to learn automatically from the environment [36,37]. Virtual organization of agents have evolved from traditional MAS architectures, they allow for the organization of groups according to their tasks within the system [38]. They also allow for solving complex sub-problems (like sensor management, data collection, information analysis or decision making [39]).

Unlike monolithic systems, agent based VO systems allow for quick and easy coupling of new agents responsible for reusing energy in other greenhouses. Thus, the use of the PANGEA framework [9] makes it possible to develop a robust, fail-safe, scalable system that allows for the increase of the number of greenhouses to which energy is distributed. Another advantage of this approach is the ability to detect potentially crucial situations, such as operating errors in the technology and to manage them as effectively as possible.

Therefore, by combining these three artificial intelligence techniques, the entire system can be designed (agent based VOs) in such way that it adapts to the environment at all times. The combination of CBR with ANN allows the system to make very accurate predictions on the basis of similar past behaviours of each greenhouse participating in the system.

## 3. System Overview

GreenVMAS is an Ambient Intelligence Organization based MAS whose purpose is to make greenhouses more profitable by recycling waste energy from power plants. First, the deployed infrastructure and the system architecture will be defined, then the AI techniques employed by the system and user software application will be described.

### 3.1. Infrastructure

GreenVMAS reuses waste energy (hot water) from power plants to heat greenhouses, its subsystems and its connections are presented in this subsection.

Sensors required by the system are deployed in this infrastructure in order to distribute the energy in an efficient way, considering the greenhouse’s requirements which are defined by the farmer through the software described in Section 3.4.

**Figure 1:**
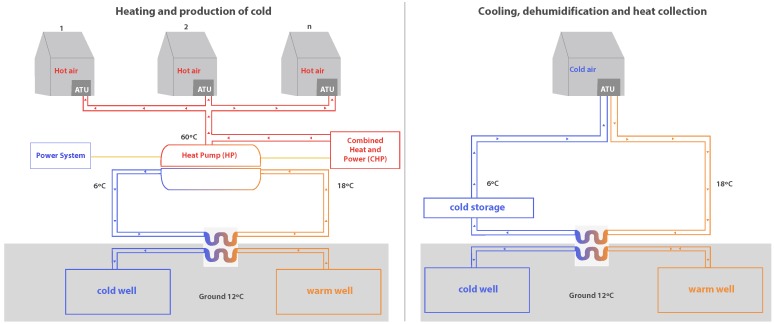
Energy Recovery Infrastructure Schema.

Figure 1 shows a diagram of the infrastructure for both the heating system and the cooling system. The main novelty of this work is shown in the heating and production of cold system. The waste energy generated by the power plant is transformed into hot water and electric power thanks to a Combined Heat and Power (CHP) module.

The generated electrical energy powers the electric Heat Pump (HP), which heats the water and distributes it to each Air Transfer Unit (ATU). The HP is also backed-up with a traditional power grid system and a photovoltaic system, which supply energy to the HP at times when the CHP is not able to fully satisfy the energy needs.

In addition to the feed, the HP receives warm water from the corresponding water tank that uses geothermal techniques to maintain its temperature at approximately 18 °C. The HP causes the temperature of the water to rise to 60 °C and this output current is mixed with the hot water current coming from the CHP, which is also at a temperature of 60 °C. As a result of the operation of the electric HP, a cold water current is also produced as an output, it is approximately 6 °C and will rise to 18 °C in the geothermal circuit which it follows to once again be the input of the HP. When the water reaches 60 °C, it is distributed to the greenhouses. The hot water first goes to the ATU where it is converted into hot air, used to maintain the target temperature in each greenhouse.

When the climatic circumstances require the addition of cold and not hot air, the circuit that is used is the one shown on the right-hand side of Figure 1. In this case, waste energy is not recovered (neither CHP nor HP are used), instead a basic geothermal system is used which is not described in this article, nor evaluated in the case studies presented below.

### 3.2. GreenVMAS Architecture

The deployed infrastructure contains valuable information inside it, this information has to be extracted in order to recycle waste energy efficiently. A heterogeneous sensor network is deployed to have a detailed view of the processes (CHP, Cold Water Tank, Hot Water Tank, Solar harmonic, ATU) that occur within the system and take the appropriate actions.

**Figure 2:**
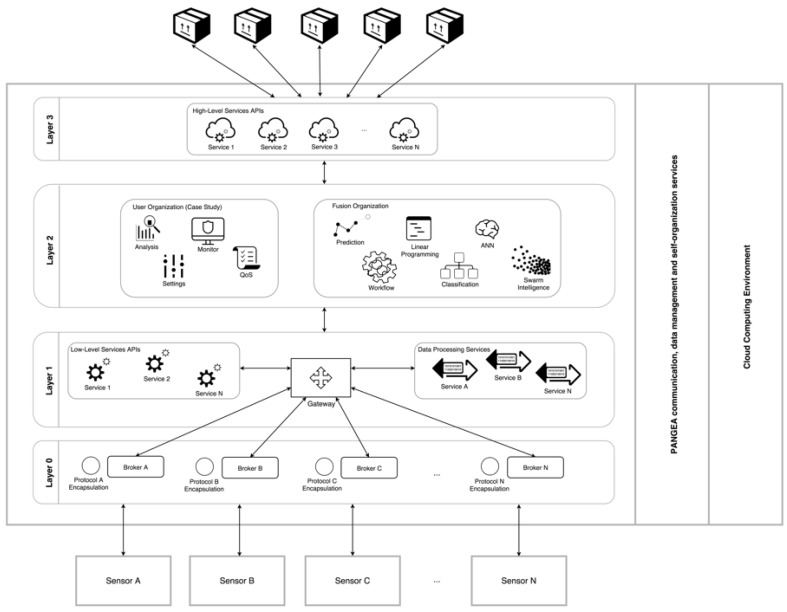
GreenVMAS architecture schema.

Data generated by the sensor layer are sent to the platform where they are processed as explained in Figure 2. The figure shows the different layers that make up the system. Information flows vertically in both directions. Two big modules that support the platform are shown on the right hand-side: PANGEA and the Cloud Computing (CC) environment.

The intelligent part of the system is responsible for achieving the desired results. This intelligent part is based on a VO MAS which is implemented in the software platform using the PANGEA framework, as presented in Figure 2. PANGEA consists of a multi-agent platform, it provides self-organization capacities through the management of an agent based VO. A complete description of PANGEA can be found in [9]. It runs on a CC environment based on OpenStack, so the storage and resources it provides are sufficient for executing the functionalities of the software platform.

This platform consist of four layers, which have a two-way relationship and are developed according to the classification of the Join Directors of Laboratories (JDL) fusion model [40]. The modular architecture of the system has two main advantages: (i) it allows to decouple the different functional parts, in this way the changes in one module do not affect the rest of the modules; (ii) modules that perform the same functionalities but use different approaches can be exchanged (e.g., two modules can apply a different methodology or different information transmission technologies to solve the same issue). Thanks to this, it is possible to execute modules according to the requirements of different stages. In addition, these modules can be executed in parallel and only the best results offered by either of the modules are considered.

Layer 0 is on the bottom of the system, its features make the system an open platform that facilitates the dynamic integration of new sensors and communication technologies. This layer acts as a broker that defines communication with multiple networks of different natures (BLE, ZigBee, Wi-Fi, 6LoWPAN, etc.) and obtains raw data from sensor networks. The process followed in Layer 0 corresponds to Level 0, “Data Assessment” of the JDL model.

Layer 1 corresponds to Level 1 of the JDL model. It is responsible for offering low-level services. The main actor in this layer is the Gateway, it processes the contextual information that Layer 0 provides to Layer 1. The Gateway allows upper and lower layers to communicate with each other by sending and receiving requests (messages) in both directions. Inside this layer, there are two VOs: one in charge of providing low-level services and the other of data processing services. When raw data are obtained, the Gateway uses adapters from the VOs to standardize the received information. The services it provides are normalization, signal filtering or other processing services. To enable this, service adapters include algorithms. An API allows upper and lower layers to interact.

Layer 2 is connected to information fusion Levels 2–4 in the JDL model. Here, the platform is structured as a MAS and it is based on a VO. Every organization performs different tasks which allow to manage the information gathered at lower levels in an intelligent way. The MAS incorporates a series of agents that have been specifically designed to interact with the low-level services provided by Layer 1. Additionally, the design of several intelligent agents specialized in information fusion are included. The cognitive part of the system is found in this layer. Two VOs represent this layer in the MAS: The user organization (directly related to the case study) and the fusion organization. The first organization contains different agents that ensure the system operates correctly, that must follow specified settings or quality parameters. The information fusion organization is the intelligent part of the system, it implements two artificial intelligence techniques: a Case-Based Reasoning (CBR) system and ANN. They allow the system to achieve its aims and will be described in more detail later in the article. These artificial intelligence techniques calculate parameters like the heat transfer coefficient of the walls of the greenhouse and the soil or the density of air and water, therefore the system does not have to perform these calculations. Parameters that affected the use case and which have been considered in the study are: solar radiation (W/m^−2^), relative humidity (%), humidity ratio ([kg/kgDA] ∗ 10^−5^), indoor air temperature (°C), outdoor air temperature (°C), cultivation area (m^2^), average height of the greenhouse, specific heat of water at the ATU input, and volume of water in heating pipes and tanks.

Finally, layer 3 is the top part of the software architecture. It is represented in the MAS by a VO that includes different high level services. These services can be accessed by the proposed control applications or even by third party applications. The VO includes different agents providing secure real time services. For example, energy efficiency services are used by interactive data analytics applications. Some of the services included in this layer offer input points that allow to setup the sensor architecture, configure the settings of every greenhouse or define energy distribution patterns. Layer 3 is connected to Level 5 and Level 6 of the JDL model and it provides one module that allows to manage and customize end user services.

### 3.3. CBR and ANN for Estimating Greenhouse Energy Requirements

To distribute the recovered energy efficiently and equitably, it is necessary to estimate the amount of energy that each greenhouse requires for proper crop growth. In the case study, the energy requirements of the greenhouses were estimated in order to maintain their indoor temperature as close as possible to the target temperature. These estimates were made in 15-min time intervals. To determine the most influential parameters, the correlation analysis and Kruskal–Wallis [41] methods were used. The Kruskal–Wallis method is used to test if a dataset originates from the same distribution, determining the dependency between the variable that is being studied and the rest of the variables.

**Figure 3:**
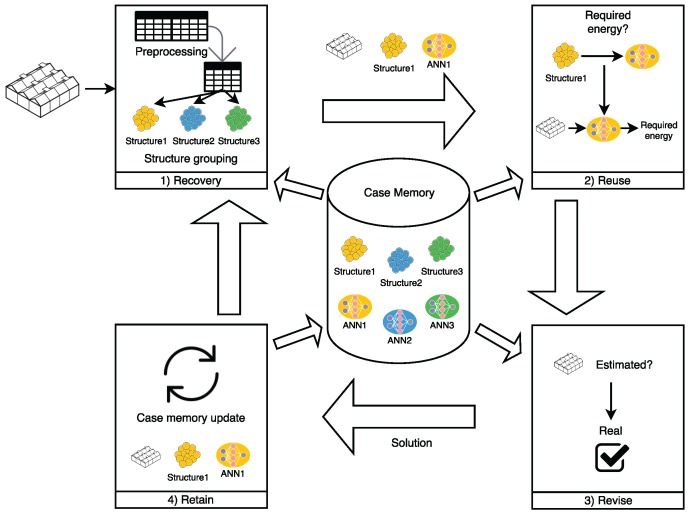
CBR cycle to predict the required energy.

Once the most influential parameters are determined, the CBR agent estimates the energy requirements, as shown in Figure 3. The case memory is grouped by the type of the greenhouses’ architectural structure. In the present case study, there were three types of structures, so there were three separate clusters in the case report. In these three clusters, the case base was organized to include the following in every case: target temperature ($temp_{t}$), outdoor temperature ($temp_{e}$), (initial) indoor temperature ($temp_{0}$), solar radiation ($s_{r}$), relative humidity (*h*), humidity ratio ($h_{r}$), ventilation (*v*), current weather station (*s*) and the amount of energy needed to change the (initial) indoor temperature to target temperature (*e*).

The developed system is to be applied in multiple scenarios. Since CBR systems are based directly on historical data [42], it is necessary to include a mechanism that will allow the CBR system used in this work to adapt to any context. To this end, an ANN is incorporated. Every cluster has a trained MLP where the inputs are target temperature ($temp_{t}$), outdoor temperature ($temp_{e}$), (initial) indoor temperature ($temp_{0}$), solar radiation ($s_{r}$), relative humidity (*h*), humidity ratio ($h_{r}$), ventilation (*v*), current weather station (*s*) and the architectural structure of the greenhouse (*g*), and the output is the prediction of the amount of energy (kWh) required to change the (initial) indoor temperature to target temperature ($e_{p}$). Thus, prediction is also part of the cases. Every case *i* includes the trained ANN for greenhouse type ($ANN_{g}$) in addition to the required information, following the structure defined in Equation (1).

(1) $${C_{g}}_{i}=\left(ANN_{g},{temp_{t}}_{i},{temp_{e}}_{i},{temp_{0}}_{i},{s_{r}}_{i},h_{i},{h_{r}}_{i},v_{i},s_{i},e_{i}\right)$$

In the recovery phase, the system recovers the trained ANN associated with the structure of the corresponding greenhouse which contains previous cases. In the reuse phase, the network is used to generate the prediction. Finally, the data and training are updated in the revise phase, once the target temperature has been reached and the required amount of energy is known.

The ANN is trained periodically, both manually and automatically. Manual training involves the use of a graphic user interface which facilitates the process. Automatic training, on the other hand, involves including a defined number of new cases in the system. In manual training, the evolution of error is analyzed with a set of training data (70% of data are used for training and 30% are used for testing). When the error starts to reduce at a slower rate, the ANN is validated with the test data. As the training continues, the error produced in the test data reduces.

The ANN configuration is as follows: nine neurons in the input layer, one neuron in the output layer. In the middle layer 2n+1 neurons are placed where *n* is the number of neurons in the input layer. This criterion is based on Kolmogorov’s theorem [43], so 19 neurons are used in the hidden layer. The activation function is the sigmoid, which is defined according to the expression shown in Equation (2).

(2) $$f\left(x\right)=\frac{1}{1+e^{-\alpha x}}$$

Concretely, α=1, it is necessary to define the functions which allow to update the *bias* and the weights of the neural network layers.

Weight updating for the weights that join the middle layer with the output layer, is defined in Equation (3) [29].

(3) $$w_{kj}^{p}\left(t+1\right)=w_{kj}^{p}\left(t\right)+\eta \left(d_{k}^{p}-y_{k}^{p}\right)\left(1-y_{k}^{p}\right)y^{p}ky_{j}^{p}+\mu \left(w_{kj}^{p}\left(t\right)-w_{kj}^{p}\left(t-1\right)\right)$$

where: $w_{kj}^{p}$ is the weight between the hidden layer’s neuron *j* and the output layer’s neuron *k*, $d_{k}^{p}$ is the pattern output value *p* for the output layer’s neuron *k*, η is the learning rate, generally η∈(0.05,0.5), μ is the near-zero moment, for example μ=0.1, $y_{k}^{p}$ is the output value of the output layer’s neuron *k*, $y_{j}^{p}$ is the output value of the hidden layer’s neuron *j*.

Weight updating for the *bias* (θ) associated with the neurons in the output layer, is defined in Equation (4).

(4) $$\theta_{k}^{p}\left(t+1\right)=\theta_{k}^{p}\left(t\right)+\eta \left(d_{k}^{p}-y_{k}^{p}\right)\left(1-y_{k}^{p}\right)y_{k}^{p}+\mu \left(\theta_{k}^{p}\left(t\right)-\theta_{k}^{p}\left(t-1\right)\right)$$

where: $\theta_{k}^{p}$ is the *bias* associated with the output layer’s neuron *k*.

Weight updating for the weights that join the input layer with the middle layer is defined in Equation (5).

(5) $$w_{ji}^{p}\left(t+1\right)=w_{ji}^{p}\left(t\right)+\eta \left(1-{\left(y_{j}\right)}^{p}\right)y_{j}^{p}\left(\sum_{k=1}^{M}\left(d_{k}^{p}-y_{k}^{p}\right)\left(1-y_{k}^{p}\right)y_{k}^{p}w_{kj}\right)x_{i}^{p}+\mu \left(w_{ji}^{p}\left(t\right)-w_{ji}^{p}\left(t-1\right)\right)$$

where: $w_{ji}^{p}$ is the weight between neuron *i* in the output layer and neuron *j* in the hidden layer, $x_{i}^{p}$ is the input value for neuron *i*.

The update in series for the *bias* associated with the middle layer neurons is defined in Equation (6)

(6) $$\theta \left(t+1\right)=\theta_{j}^{p}\left(t\right)+\eta \left(1-y_{j}^{p}\right)y_{j}^{p}\left(\sum_{k=1}^{M}\left(d_{k}^{p}-y_{k}^{p}\right)\left(1-y_{k}^{p}\right)y_{k}^{p}w_{kj}\right)+\mu \left(\theta_{j}^{p}\left(t\right)-\theta_{j}^{p}\left(t-1\right)\right)$$

where: $\theta_{j}^{p}$ is the *bias* of neuron *j* in the hidden layer.

Once the energy requirements of each greenhouse within the system are estimated, equitable distribution is ensured by assessing overall energy needs.

### 3.4. Control and Monitoring Software Application

**Figure 4:**
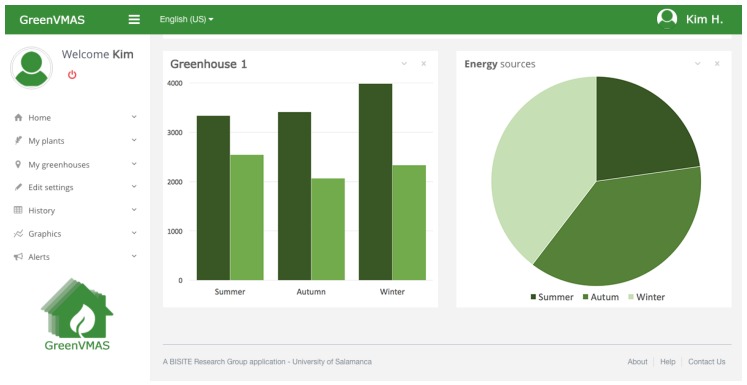
Final user software web application: in this case charts display performance and energy resources.

Figure 4 shows a screenshot of the final user application (for greenhouse owners). It provides a responsive website, where the user can both monitor their greenhouse in real time (temperature, humidity, system performance, expenditure reports, etc.) and establish the desired greenhouse conditions like temperature and humidity or adjust the parameters to the budget. All these parameters can be configured by the farmer at specific times. The system distributes the recovered energy by following patterns that the farmer predetermined and maximizing energy savings. In addition, the system generates different statistics and budgets related to production costs. It evaluates the profit obtained from using the developed system.

## 4. Case Study

This section details all the components that were involved in the case study and how the case study was conducted.

### 4.1. Deployed Sensor and Actuator Network

Sensors deployed in the infrastructure are grouped into different networks. M2M techniques were adapted to allow networks to communicate with each other. All the sensors used were static position sensors and were powered by the electrical network. In our case, the use of wired sensors (which do not need batteries and will not stop functioning unexpectedly) is more convenient than wireless technologies. On the other hand, data transmission uses wireless technology with the associated advantages when deploying this kind of communication networks. To ensure effective control of the developed system, BLE, Wi-Fi and 6LoWPAN based networks were used to connect all the sensors and actuators. 25+8×n sensors have been used for the monitoring task (where *n* is the number of greenhouses that took part in the case study and 25 is the number of sensors that the system required). Six different types of sensors were used, they are outlined in Table 2.

In addition to the sensors, a series of valves regulated different parts of the system. More specifically, one valve controlled the gas input in the CHP and 8+n valves were used to control the flow of water at the different stages in the system, where n is the number of greenhouses that were included in system. This ensured that the system was able to reach the desired result autonomously by regulating all the flows.

### 4.2. Power Plant

**Figure 5:**
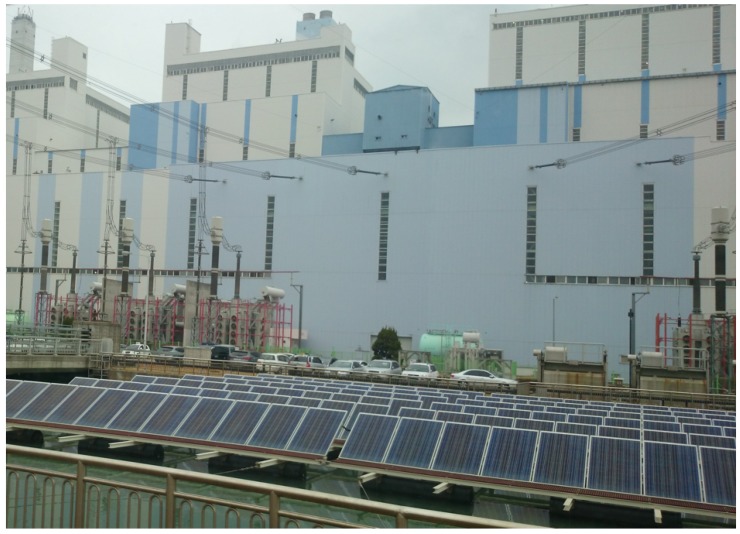
Part of the infrastructure of the Dangjin power station.

In this case study, the functionality of the system will be analyzed using information provided by Dangjin power plant, in Korea (Figure 5) was used to evaluate the performance of the system. The Dangjin complex comprises eight power plants that generate a total of 4000 megawatt (MW), 500 MW each. The proposed system was tested with the data of just one of the towers, however the complex is currently undergoing a phase of expansion and the possibility of deploying this architecture is being considered.

### 4.3. Greenhouses

The six greenhouses chosen for the case study all have an area of (300 m^2^) and all grow tomatoes. They are equipped with a Booster Bov-500 580 kW vacuum boiler system, which has 75 kW heaters. The tomato crop has specific nutritional requirements. It needs abundant watering (the leaves should not get wet as this may cause the plant to get sick). It grows in high temperatures, the roots have to be kept hot and the plant has to be exposed to direct sunshine for six to eight hours daily. Optimal temperature during the day is between 20 °C and 27 °C and at night between 13 °C and 30 °C, the temperature of the substrate must be between 15 °C and 20 °C, CO_2_ between 1000 and 2000 ppm and relative humidity between 55% and 60%. The crop is transplanted several times during its growth until it is two months old when it no longer requires transplantation. At this time the optimal temperature for the plant to mature is 30 °C by day and 17 °C by night. For this reason, the greenhouses that are part of the case study are configured differently, to check what conditions are allow to obtain a high quality product after three months when the fruit is in the harvesting phase. Tomatoes are climacteric fruit, this means that they are picked when still green and ripen outside the tomato vine. During transport they are often stored together with apples or other tomatoes, as they release ethylene gas which accelerates their ripening.

**Figure 6:**
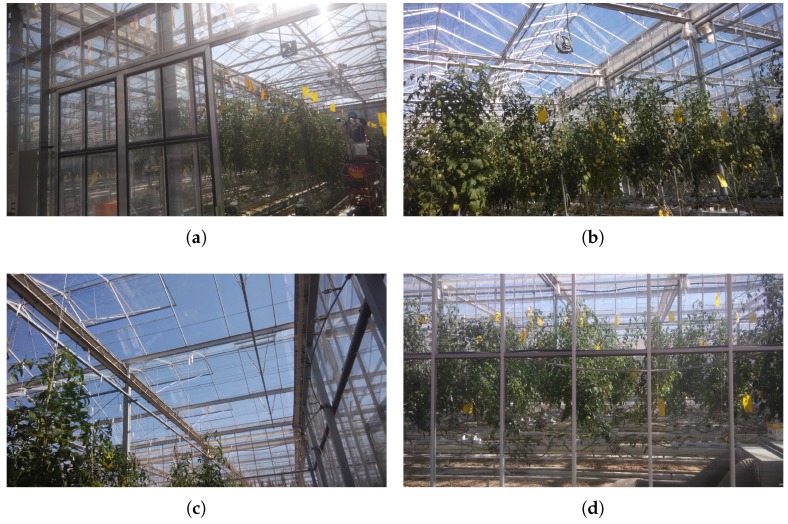
Case study greenhouse with tomato crop. (**a**) A-frame architecture; (**b**) Force fans for ventilation; (**c**) Natural ventilation; (**d**) Heater and evaporative cooling system.

Figure 6 shows the A-frame greenhouse with natural ventilation, force fans, a heater and evaporative cooling system. The case study greenhouses have different architectural structures and technical characteristics, Table 3 outlines the characteristics of each greenhouse.

### 4.4. Experimental Set-Up

The experiment was divided into two stages, a baseline stage and an implementation stage, each stage had a duration of nine months (a new crop was grown each season). Specifically, the baseline period lasted from 1 June 2015 to 29 February 2016, during this time the grid electricity consumed by the greenhouses was simply monitored. The implementation stage lasted from 1 June 2016 to 28 February 2017, energy consumption levels were measured during this time with the implemented system. The measurements collected over the two periods were later compared in order to evaluate the performance of the developed system and its efficacy in decreasing energy consumption levels. The tests were carried out in the same seasons, thus the measurements that were compared had been obtained in similar climatic conditions, ensuring fair results. In the designed case study, the structure of all the greenhouses was different and this impeded us from collecting energy consumption data in one year (since this meant each greenhouse had different energy needs). This would have been possible if we had two, architecturally equal greenhouses, as one greenhouse would have acted as a control and the other would have measured the performance of the proposed system.

When calculating the climate conditions required by each greenhouse ($Q_{cli}$), the conditions outside the greenhouse were considered in order to establish the optimal climate inside the greenhouse. The main parameters that characterize the climate and have been included in the case study are: Maximum solar radiation intensity, Outdoor temperature and humidity, and Wind direction and average wind speed. The parameter that had the most influence on the energy balance of a greenhouse was outdoor temperature, which directly determines the cooling and heating needs.

Cooling Energy Requirements (CER) where calculated for each greenhouse in the following conditions in order to avoid evapotranspiration: summer day at noon, with maximum solar radiation and a newly transplanted crop. To calculate the Heating Energy Requirements (HER) of each greenhouse, data were collected on a winter day at 7 a.m., with no solar radiation and a two month old crop (evapotranspiration occurs at this stage). The results of these measurements are shown in Table 4.

The factors involved in the balance of energy in a greenhouse are expressed in the form of energy intensity. According to the first law of thermodynamics, the energy obtained by the system is balanced with the energy lost by it. Although each author considers a series of different parameters, we defined energy balance with the following equation:

$$SR+Q_{cli}=Q_{cc}+Q_{ren}+Q_{evp}+Q_{soi}l\left(W\right)$$

where:

SR: Solar radiation
$Q_{cli}$: Heat energy to be supplied to (Qcal) or removed (Qref) from the greenhouse
$Q_{cc}$: Heat loss due to conduction-convection
$Q_{ren}$: Sensitive and latent heat loss due to indoor air changes $Q_{evp}$: Latent heat consumed in plant and soil evotranspiration
$Q_{soil}$: Heat flow loss due to conduction to the ground

The first part of the equation corresponds to the obtained energy and the second part corresponds to the energy lost through the greenhouses’ ventilation vents. Since the energy needs of the greenhouse depend fundamentally on the difference between the indoor and outdoor temperature, i.e., the temperature that is to be maintained inside the greenhouse (ideal temperature for the tomato crop) and by how much this temperature differs from the outside temperature.

To determine the air-conditioning needs of each greenhouse, the equation for energy balance was used in the three cases (summer, autumn and winter). In summer it was necessary to cool down and in winter to heat up the greenhouse, due to the difference between indoor and outdoor temperature.

Once the energy needs of each greenhouse were determined, the agents from the virtual organization based Platform were coordinated in order to fulfil their roles in the system: the acquisition of sensor data, execution of the IF algorithm, valve opening/closing, and execution of the CBR agent cycle to obtain the amount of energy necessary to meet the needs of each greenhouse. The results of the conducted case studies for the different seasons of the year are outlined below.

## 5. Results

This section assesses whether the results obtained in the case studies confirm the hypothesis posed in this work. The hypothesis that motivated this work stated that it is possible to reuse the energy waste that is generated by a power plant to heat greenhouses, in this way reducing the energy supplied by the traditional power grid. The following subsection describes the conducted case studies and the results; the consumption levels before and after the implementation of the system verify our hypothesis.

By introducing hot or cold air into the greenhouse, the temperature of the greenhouse is raised or lowered, this means that less energy is needed from the power grid to maintain an optimal temperature in the greenhouse. Thus, the results of the three case studies are expressed in kWh before and after the implementation of the platform.

To verify our hypothesis Student’s *t*-test and Levene’s test were performed. They assessed the difference of means (electrical consumption in kWh) and variances between the data obtained before and after the implementation of the platform. In the tests, the level of α was established as 0.05 and the size of the samples was n1 = 90, n2 = 90, which was equivalent to the number of days over which the crops grew, where F is Levene’s Test for equality of variances and *t* is the *t*-test for equality of means [44]. The established hypothesis H0 stated that—the mean energy consumed by the greenhouse before the implementation of the system is equal to the mean energy consumed by the greenhouse with the GreenVMAS system; H1 stated that—the average energy consumption in the greenhouse is lower when the proposed GreenVMAS is implemented. If the *p*-value is less than 0.05 (significance value) than hypothesis H0 is rejected and H1 is proved true, thus the system consumes less energy from the grid by using the energy from the power plant. In all cases, except greenhouse 5, the percentage of use after the platform had been implemented was notably lower, as the *p*-value was always under 0.05. In conclusion, the proposed VO platform greatly contributes to energy savings in greenhouses.

### 5.1. Energy Consumption in Summer

In the summer, the optimal temperature for a tomato cultivation is 25 °C and the maximum humidity is 70%. The summer in Dangjin is warm. In the morning, outside temperature increases and humidity decreases. The outside temperature is not high enough in the mornings, as a result heating needs to be turned on in the greenhouse in order to achieve the target temperature. At midday, the temperatures rise and are too high for the crop, as a result it is necessary to cool the air in the greenhouse.

The CBR agent executes a cycle in which it retrieves the cases from the cluster that is related to the architectural structure of the greenhouse (A-frame, Quonset and Arch). This section presents the case of greenhouse 1, so the CBR cycle is associated with the A-frame cluster and the input parameters: target temperature, outdoor temperature, indoor temperature (initial), solar radiation, relative humidity, humidity ratio, ventilation, current weather station and the amount of energy required normally to raise the initial indoor temperature to target temperature (without the system). Table 5 outlines the input parameters collected by the deployed sensor infrastructure.

Table 6 shows the results of the Student’s *t*-test and Levene’s test. The level of consumption is notably lower after the implementation of the platform, with *p*-value always under 0.05, proving that the hypothesis is true.

### 5.2. Energy Consumption in Autumn

Autumn temperatures in Dangjin are lower than in the summer but since the climate is warm the difference between minimum and maximum temperatures is not large. However, it was necessary to heat the greenhouses at all times because the outside temperature was lower than the target temperature (26 °C). Table 7 shows how the difference between outdoor and indoor temperature is small, but energy is required to achieve target temperature. Energy is also required to reduce humidity in the greenhouse, the tomato crop requires (less than 70%).

The CBR agent executes the CBR cycle in which it retrieves cases with the architectural structure of each greenhouse (A-frame, Quonset and Arch) from the associated cluster. The CBR agent provides the cases associated with the autumn season, since the input variables (target temperature, outdoor temperature, indoor temperature (initial), solar radiation, relative humidity, humidity ratio, ventilation, current weather station and the amount of energy for temperature change) have similar values. From all the cases that the CBR agent retrieves, the system automatically selects the one that best fits the input variables. Once executed, the case is stored in the case database, with the adapted changes.

As we can see in Table 8, results of the Student’s *t*-test and Levene’s test performed to assess the difference of means and variances between before the platform usage data and the data collected during the use of the platform in autumn season. In all cases, except greenhouse 5 again, the percentage of use after the platform use is notably lower, with *p*-value always under 0.05, which shows that there is energy saving thanks to the VO platform. The *p*-value results are minimally worse than in the summer season; this is because the minimum temperatures cause a greater difference from the temperature required by tomatoes. However, the results show that energy savings are evident.

### 5.3. Energy Consumption in Winter

Winter temperatures in Dangjin are characterised by a minimum temperature of less than 0 °C, these conditions do not allow the tomato plant to grow outside the greenhouse. The temperature at which the crop stops to grow is 12 °C, so the difference between the outside temperature and the minimum growth temperature is high. However, if the crop is to grow in optimal conditions, the ideal temperature in the greenhouse would be between 20 °C to 27 °C during the day. In the night, the temperature can range between 13 °C and 30 °C due to lower solar radiation. In order to prevent excess energy consumption, the target temperature in this case is set at 24 °C, as shown in Table 9.

In Table 10 we can observe that in winter the difference in the energy consumed before and after the implementation of our system is greater than in the other two case studies (summer and autumn), this is because more energy was required and our system supplied it from the power plant producing greater energy savings. As in the previous case studies, the *p*-value also shows a high degree of acceptance of the system’s functionality ( *p*-value is lower than 0.05).

## 6. Conclusions and Future Work

This paper presented an innovative approach to reusing energy waste from nuclear energy plants. It employs an agent based virtual organization to reduce the levels of energy greenhouses required to maintain the crops in optimal conditions. The use of an agent system in this energy management problem was fundamental; it allowed us to monitor the state of the crop and coordinate the opening and closing of the valves which release hot water in the greenhouse. This architecture based on virtual agent organizations complied with the requirements posed by the hypothesis (that waste energy from a power plant can be reused in greenhouses to achieve energy savings), as its features enabled sensor data collection, information fusion, information processing and decision making.

Agent-based virtual organizations use data from the sensor network which was deployed in the power plant and the greenhouses used in the case study. From the obtained test results, we can see that the use of a VO MAS system was essential for recovering waste energy and saving energy and money on the heating of greenhouses. As a result of reduced energy consumption, farmers achieve economic savings and the level of CO_2_ emissions is lower. The CBR system made it possible to obtain the energy that was necessary to achieve optimal temperature for crop growth. Energy requirements were established on the basis of various parameters: solar radiation, humidity, outdoor and indoor air temperature, and soil temperature. The combination of the ANN and the CBR creates context awareness; this allows the system to adapt quickly to new cases and predict energy requirements accurately even if new greenhouses or sensors appear in the system.

From the case study results, we can observe that the system’s performance was at its best during winter. As described in the results section, in this period more energy is needed in order to keep the temperature that will not put the tomato plant at risk of getting damaged and will allow it to grow properly. Significant reductions were achieved in the three case studies. In the summer case study, consumption was decreased by 23.79% in autumn by 39.40% and in winter 41.46%. Since autumn temperatures in 2017 were much lower than in previous years, the percentage of energy saved was high, almost the same as in winter. Obtaining average savings of 34.88%.

One of the conclusions drawn from the conducted case studies is that the amount of water transpired by plants is proportional to the amount of solar radiation they receive. This can be used to cool the greenhouse environment because plants are used as evaporative cooling surfaces. The water that plants transpire reduces the temperature of the leaves. The sensitive heat of the air is removed by the ventilation system; this reduces the temperature in the greenhouse.

Therefore, it is necessary to evaluate how long it takes for the investment in the infrastructure and its deployment in the power plant to pay-off. Future lines of research will be concerned with incorporating energy distribution algorithms as opposed to the current equitable distribution, since with this equation some greenhouses may receive more energy than others.

References

## Figures and Tables

**Table 1:** Summary of proposals for waste energy recovery in other projects.

	Common Elements	Differentiating Elements	Advantages	Drawbacks
GASTone	Energy recovery and storage.	Recovery of kinetic energy through a belt driven generator and waste heat with an energy cascading approach.	Vehicle efficiency well above 50% at an acceptable cost.	Requires engine modification for the adoption of this solution.
TRIPOD	Optimization of energy consumption thanks to energy recovery.	Integration of podded propulsion and tip loaded endplate propellers in combination with energy recovery based on counter-rotating propeller (CRP) principle.	High potential in fuel savings and emission reductions	The main propeller is driven by a 2-stroke engine which produces high emission.
HEATRECAR	Efficient use of the energy wasted in the form of heat in thermal engines.	The power module converts heat energy directly into electricity.	Reduction of fuel consumption and abatement of CO_2_ emissions due to the reduced mechanical load at the crankshaf.	Conversion of a exhaust gases into electricity, but there is no reduction of electricity consumption.
INTHEAT	Heat recovery using heat transfer units.	Method for dealing with the main exchanger geometry details in HEN retrofit problems.	Energy consumption in crude oil distillation could be decreased by 30% using the heat recovery system.	FT values increase the computation difficulty for optimizing HEN retrofit problems.

**Table 2:** Sensors used in the system.

Sensor Type	Function	Position	Number
Thermal	It measures the temperature of the water or the air in the point of the system where it is placed.	-2 inputs (gas and water) and water output of the CHP module.	3
		-Input and output (water) of the Hot Water Tank module.	2
		-Input and output (water) of the Cold Water Tank module.	2
		-Input and output (water) of the Heat Pump module.	2
		-Input and output (water) of each ATU module.	2 × *n*
		-Air inside of every greenhouse.	1 × *n*
		-Outdoor temperature.	1
Flowmeter		-Gas input and water output of the CHP module.	2
	2 different models are used:	-Output (water) of the Hot Water Tank module.	1
	-Measurement of the amount of gas flowing through a point of the system.	-Output (water) of the Cold Water Tank module.	1
	-Measurement of the amount of water flowing through a point of the system.	Output (water) of the Heat Pump module.	1
		-Input and output (water) of each ATU module.	2 × *n*
Manometer		-Gas input and water output of the CHP module.	2
	2 different models are used:	-Output (water) of the Hot Water Tank module.	1
	-Measurement of the pressure of gas in a point of the system.	-Output (water) of the Cold Water Tank module.	1
	-Measurement of the pressure of water in a point of the system.	-Output (water) of the Heat Pump module.	1
		-Output (water) of each ATU module.	1 × *n*
Ammeter	This kind of sensor measures the current in a point of the electric part of the system.	-Output of the CHP module.	1
		-Output of the Solar Farm module.	1
		-Input of the Heat Pump module.	1
		-Every ATU module	1 × *n*
Volumetric	This kind of sensor measures the volume occupied by the water of the tanks.	-Hot Water Tank module.	1
		-Cold Water Tank module.	1
Hygrometer	This kind of sensor measures the humidity of a greenhouse.	-Air inside of every greenhouse.	1 × *n*

**Table 3:** Characteristics of the greenhouses.

	Greenhouse 1	Greenhouse 2	Greenhouse 3	Greenhouse 4	Greenhouse 5	Greenhouse 6
Floor area	300 m^2^	300 m^2^	300 m^2^	300 m^2^	300 m^2^	300 m^2^
Structure	A-frame	A-frame	A-frame	Quonset	Arch	Arch
Glazing	Rigid Plastic (Polycarbonate) Double Layer	Rigid Plastic (Polycarbonate) Double Layer	Rigid Plastic (Polyethylene) Double Layer	Glass Single Layer	Rigid Plastic (Polyethylene) Double Layer	Rigid Plastic (Polycarbonate) Single Layer
Ventilation	Forced 2 fans × 60	Forced 2 fans × 60	Forced 2 fans × 60	Natural (passive) 1 Inlets × 30	Forced 2 fans × 60	Natural (passive) 1 Inlets × 30
Evaporative cooling	14 wet pads	14 wet pads	7 wet pads	7 wet pads	14 wet pads	7 wet pads
Shade curtains	No	No	Yes 30% Light reduction	Yes 30% Light reduction	Yes 30% Light reduction	No
Vacuum boiler	1 Booster Bov-500	1 Booster Bov-500	1 Booster Bov-500	1 Booster Bov-500	1 Booster Bov-500	1 Booster Bov-500
Heating	2 Heaters	2 Heaters	2 Heater	1 Heater	1 Heater	1 Heater

**Table 4:** Measurements to calculate the energy needs of each greenhouse.

	Greenhouse 1		Greenhouse 2		Greenhouse 3		Greenhouse 4		Greenhouse 5		Greenhouse 6	
	CER	HER	CER	HER	CER	HER	CER	HER	CER	HER	CER	HER
Max. solar radiation intensity (W/m^2^)	534	1	536	1	566	1	783	2	500	1	572	1
Out. temperature (°C)	22.9	−13	23.6	−13.4	23.1	−13.2	23.97	−12.7	22.8	−12.4	23.7	−12.9
Out. humidity (%)	66	78	66	78	65	78	66	78	58	77	66	74
Wind direction	W	E	WE	E	WE	E	WE	E	WE	E	WE	E
Avg. wind speed (km/h)	7	13	6.7	12	6.6	11	5.3	11	6	7	7	10

**Table 5:** Climate characteristics on a day in June (Summer) tomato cultivation in Greenhouse 1.

Time	Out. Temp (°C)	In. Temp (°C)	Target Temp (°C)	Soil Temp (°C)	Out. SR (W/m^2^)	In. SR (W/m^2^)	Out. RH (%)	In. RH (%)
10:00	21.6	22.5	25	18.5	533	357.9	72	72.5
10:15	21.5	22.5	25	18.5	573	384.8	72	72.5
10:30	22.2	23.1	25	18.6	615	413	72	72.3
10:45	21.9	23	25	18.7	636	427.1	72	72.3
11:00	22.5	23.4	25	18.8	663	445.2	66	68.3
11:15	22.4	23.5	25	18.9	699	469.4	66	67.1
11:30	22.4	23.5	25	19	566	380.1	66	66.7
11:45	23.1	24	25	19	566	380.1	66	66.5
12:00	22.9	24	25	19.1	534	358.6	66	66.4

**Table 6:** Results of the Student’s *t*-test and Levene’s test performed on a summer tomato cultivation. These tests were performed to assess the difference of means (electrical consumption in kWh) and variances between the data obtained before and after using GreenVMAS.

	Before GreenVMAS		After GreenVMAS					
	Mean (kWh)	Stdr. Deviation (kWh)	Mean (kWh)	Stdr. Deviation (kWh)	*t*	*p*-Value (2-tailed)	F	*p*-Value
Greenhouse 1	3181.57	1109.49	1905.50	1628.49	6.143	0.000	27.088	0.000
Greenhouse 2	3315.73	1087.10	1917.76	1617.96	6.804	0.000	29.163	0.000
Greenhouse 3	3313.36	1073.52	2585.14	1749.02	3.366	0.001	32.797	0.000
Greenhouse 4	3450.92	1212.23	2924.43	1500.65	2.589	0.010	4.692	0.032
Greenhouse 5	3441.22	1191.39	3164.98	1431.15	1.407	0.161	1.804	0.181
Greenhouse 6	3315.71	1075.01	2757.86	1457.52	2.922	0.004	8.791	0.03

**Table 7:** Characteristics of the climate on a day in November (Autumn) tomato cultivation in Greenhouse 1.

Time	Out. Temp (°C)	In. Temp (°C)	Target Temp (°C)	Soil Temp (°C)	Out. SR (W/m^2^)	In. SR (W/m^2^)	Out. RH (%)	In. RH (%)
1:00	4.9	5.7	26	9.6	2	1.3	74	84
1:15	4.7	5.5	26	9.6	2	1.3	74	84.2
1:30	4.3	5.1	26	9.5	2	1.3	74	84.5
1:45	4.2	5	26	9.5	2	1.3	74	84.7
2:00	3.9	4.7	26	9.5	2	1.3	74	85
2:15	3.9	4.6	26	9.5	2	1.3	74	85
2:30	3.7	4.5	26	9.4	2	1.3	78	87.8
2:45	3.6	4.3	26	9.4	2	1.3	78	88.8
3:00	3.4	4.2	26	9.4	2	1.3	78	89.2

**Table 8:** Results of the Student’s *t*-test and Levene’s test performed in autumn tomato cultivation. Difference of means (electrical consumption in kWh) and variances between the data obtained before and after using GreenVMAS.

	Before GreenVMAS		After GreenVMAS					
	Mean (kWh)	Stdr. Deviation (kWh)	Mean (kWh)	Stdr. Deviation (kWh)	*t*	*p*-Value (2-Tailed)	F	*p*-Value
Greenhouse 1	3546.46	1102.56	1526.11	2016.08	8.341	0.000	10.961	0.001
Greenhouse 2	3502.21	1045.66	2165.59	7793.05	1.634	0.104	3.031	0.083
Greenhouse 3	3545.18	1095.84	3068.74	768.16	17.555	0.000	21.581	0.000
Greenhouse 4	2896.62	735.75	2436.29	1835.43	2.208	0.028	107.806	0.000
Greenhouse 5	3481.08	1213.72	1260.57	1273.71	11.973	0.000	0.546	0.461
Greenhouse 6	3493.47	1090.14	1942.86	1132.80	15.391	0.000	4.088	0.045

**Table 9:** Weather characteristics on one day in January (winter) tomato crop in Greenhouse 1.

Time	Out. Temp (°C)	In. Temp (°C)	Target Temp (°C)	Soil Temp (°C)	Out. SR (W/m^2^)	In. SR (W/m^2^)	Out. RH (%)	In. RH (%)
14:00	−6.4	−5.8	24	−9.1	365	245.1	72	96.9
14:15	−6.3	−5.6	24	−9.1	295	198.1	64	93.7
14:30	−6.7	−5.9	24	−9.1	252	169.2	64	93.5
14:45	−6.2	−5.6	24	−9.1	335	225	65	93.6
15:00	−6.5	−5.7	24	−9.1	225	151.1	65	92.5
15:15	−6.3	−5.6	24	−9.1	254	170.6	65	92.1
15:30	−6	−5.4	24	−9.1	285	191.4	65	89.9
15:45	−5.8	−5.1	24	−9.1	290	194.7	65	90.2
16:00	−5.9	−5.2	24	−9.1	216	145	65	89.6

**Table 10:** Results of the Student’s *t*-test and Levene’s test conducted on the tomato crop in winter. These tests were performed to assess the difference of means (electrical consumption in kWh) and variances between the data obtained before and after using GreenVMAS.

	Before GreenVMAS		After GreenVMAS					
	Mean (kWh)	Stdr. Deviation (kWh)	Mean (kWh)	Stdr. Deviation (kWh)	*t*	*p*-Value (2-Tailed)	F	*p*-Value
Greenhouse 1	4073.51	1154.57	1948.81	1677.09	9.900	0.000	24.285	0.000
Greenhouse 2	3918.43	1092.21	1971.77	1719.95	9.064	0.000	35.472	0.00
Greenhouse 3	4032.02	1087.68	2540.56	1600.79	7.311	0.000	22.070	0.000
Greenhouse 4	3883.44	1328.48	1328.48	1233.32	8.215	0.000	4.931	0.028
Greenhouse 5	4215.73	1113.79	3260.05	1415.91	5.033	0.000	2.975	0.086
Greenhouse 6	3799.57	1062.45	2953.60	1511.05	4.345	0.000	18.345	0.000
